# (*E*)-3-[(Di­methyl­amino)­methyl­idene]-4-phenyl-1-(prop-2-yn­yl)-1*H*-1,5-benzodiazepin-2(3*H*)-one

**DOI:** 10.1107/S1600536813032960

**Published:** 2013-12-11

**Authors:** Mohamed Loughzail, Abdesselam Baouid, Filipe A. Almeida Paz, José A. S. Cavaleiro, El Hassane Soumhi

**Affiliations:** a Laboratoire de Chimie Moléculaire, Département de Chimie, Faculté des Sciences-Semlalia, BP 2390, Université Cadi Ayyad, 40001 Marrakech, Morocco; b Department of Chemistry, University of Aveiro, CICECO, 3810-193 Aveiro, Portugal; c Department of Chemistry, University of Aveiro, QOPNA, 3810-193 Aveiro, Portugal; d Equipe de Chimie des Matériaux et de l’Environnement, FSTG–Marrakech, Université Cadi Ayyad, Bd Abdelkrim Khattabi, BP 549, Marrakech, Morocco

## Abstract

The title compound, C_21_H_19_N_3_O, exhibits an *E* configuration with respect to the C=C bond between the benzodiazepine and tri­methyl­amine groups. The seven-membered diazepine ring displays a boat conformation. In the crystal, mol­ecules are linked by a C—H⋯O hydrogen bond, forming a chain along [110].

## Related literature   

For benzodiazepine derivatives, see: Di Braccio *et al.* (2001 ▶); Pevarello *et al.* (1993 ▶). For related structures, see: Loughzail *et al.* (2011 ▶); Boudina *et al.* (2006 ▶).
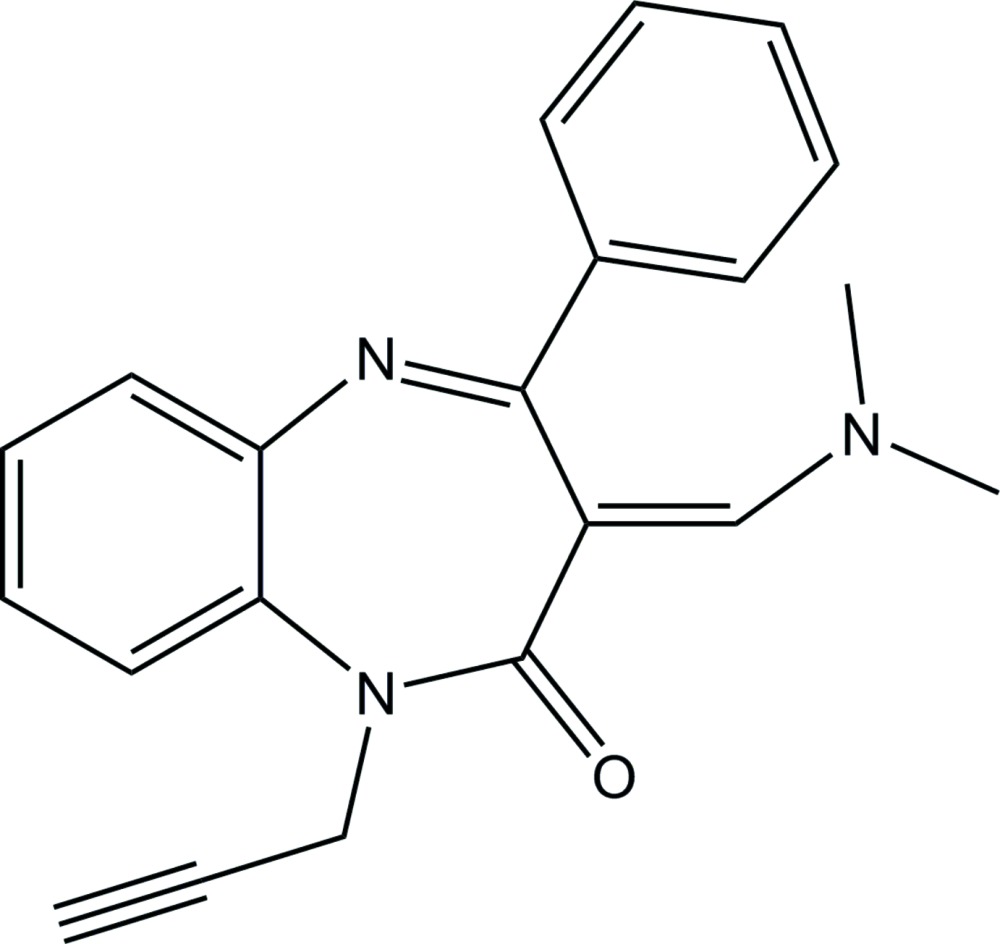


## Experimental   

### 

#### Crystal data  

C_21_H_19_N_3_O
*M* *_r_* = 329.39
Monoclinic, 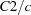

*a* = 12.8122 (10) Å
*b* = 13.9317 (12) Å
*c* = 19.7795 (14) Å
β = 94.647 (3)°
*V* = 3519.0 (5) Å^3^
*Z* = 8
Mo *K*α radiation
μ = 0.08 mm^−1^
*T* = 300 K
0.13 × 0.10 × 0.08 mm

#### Data collection  

Bruker X8 KappaCCD APEXII diffractometer
Absorption correction: multi-scan (*SADABS*; Sheldrick, 1996 ▶) *T*_min_ = 0.990, *T*_max_ = 0.994
21544 measured reflections
3220 independent reflections
2555 reflections with *I* > 2σ(*I*)
*R*_int_ = 0.032

#### Refinement  

*R*[*F* ^2^ > 2σ(*F* ^2^)] = 0.038
*wR*(*F* ^2^) = 0.099
*S* = 1.02
3220 reflections
228 parameters
H-atom parameters constrained
Δρ_max_ = 0.16 e Å^−3^
Δρ_min_ = −0.14 e Å^−3^

### 

Data collection: *APEX2* (Bruker, 2007 ▶); cell refinement: *SAINT* (Bruker, 2005 ▶); data reduction: *SAINT*; program(s) used to solve structure: *SHELXS97* (Sheldrick, 2008 ▶); program(s) used to refine structure: *SHELXL97* (Sheldrick, 2008 ▶); molecular graphics: *ORTEP-3 for Windows* (Farrugia, 2012 ▶); software used to prepare material for publication: *WinGX* (Farrugia, 2012 ▶).

## Supplementary Material

Crystal structure: contains datablock(s) I, HasRB. DOI: 10.1107/S1600536813032960/is5319sup1.cif

Structure factors: contains datablock(s) I. DOI: 10.1107/S1600536813032960/is5319Isup2.hkl

Supporting information file. DOI: 10.1107/S1600536813032960/is5319Isup3.cml

Additional supporting information:  crystallographic information; 3D view; checkCIF report

## Figures and Tables

**Table 1:** Hydrogen-bond geometry (Å, °)

*D*—H⋯*A*	*D*—H	H⋯*A*	*D*⋯*A*	*D*—H⋯*A*
C15—H15⋯O1^i^	0.93	2.50	3.415 (2)	170

Symmetry code: (i) 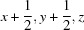.

**Fig. 1.**
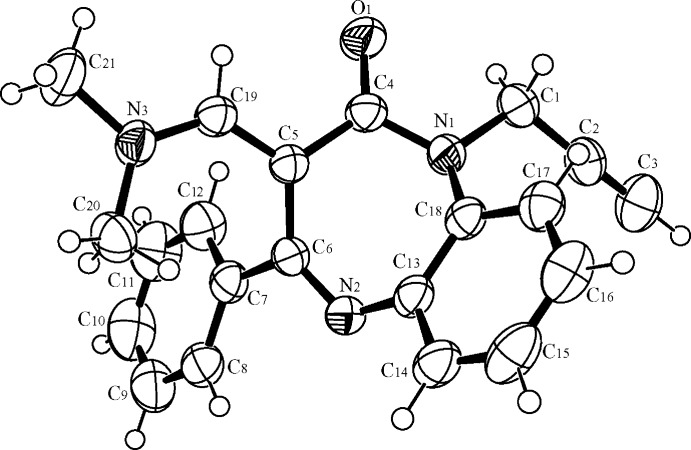
The molecular structure of the title compound with 50% probability ellipsoids.

## supplementary crystallographic information

### 1. Comment

The benzodiazepine nucleus is extremely important, as it is the base of several drugs and other biologically active compounds with different properties. A large number of structurally modified benzodiazepines have been prepared and evaluated concerning their biological activity (Di Braccio *et al.*, 2001; Pevarello *et al.*, 1993) and may be considered support for the synthesis of more active heterocyclic systems. In this class of compounds, our research team is interested in the synthesis of novel benzodiazepines derived (Loughzail *et al.*, 2011; Boudina *et al.*, 2006). Here we wish to report the synthesis *via* phase transfer catalysis and the crystallographic studies of the title compound. The title compound was prepared by action of propargyl bromide with 3-dimethylaminomethylene-4-phenyl-1,3-dihydro-2*H*-1,5-benzodiazepin-2-one using a catalytic amount of benzyltriethylammonium chloride (TBA-Cl) and sodium hydroxide aqueous solution in benzene. The obtained compound was typically characterized by ^1^H, ^13^C NMR, IR and mass spectroscopy, and the stereochemistry (*E*) of the benzodiazepine was determined by X-ray diffraction. The main geometric feature of the title compound is in good agreement with that observed in a similar compound (Loughzail *et al.*, 2011).

### 2. Experimental

A mixture of 0.6 g (2.06 mmol) of 3-[(dimethylamino)methylene]-4-phenyl-1,3-dihydro-2*H*-1,5-benzodiazepin-2-one, 0.26 g (1.14 mmol) of benzyltriethylammonium chloride and 3 ml of a 50% sodium hydroxide aqueous solution in benzene (25 ml) was stirred at ambient temperature. After 15 min, propagyl bromide was added slowly. After 8 h of stirring at 298 K, the reaction mixture was diluted with water (30 ml). The organic layer was extracted with benzene (3× 10 ml), dried over anhydrous sodium sulfate and evaporated under vacuum. The title compound was isolated by column chromatography on silica gel using hexane/ethyl acetate as eluent. The solid product was recrystallized in dichloromethane to give yellow crystals of the title compound.

Yield: 93%, m.p. 488–490 K. ^1^H NMR (300 MHz, CDCl_3_): δ 2.36 (t, J = 2.27 Hz, 1H, HC≡C), 2.58 (s, 6H, (CH_3_)_2_N), 4.21 and 4.29 (AB system, d, J = 17.9 Hz, 2H, N—CH_2_—C), 6.46 (s, 1H, C=CH—N), 7.15–8.11 (9*H*, Ar—H). ^13^C NMR (75 MHz, CDCl_3_): δ 37.1 (1 C, N—CH_2_—C), 42.6 (N(CH_3_)_2_), 71.9 (1 C, HC≡C), 78.5 (1 C, HC≡C), 97.8 (C-3), 120.9–140.9 (Ar—C, =CH), 164.8 (1 C, Ph—C=N), 169 (1 C, CO).

### 3. Refinement

All H-atoms were located in a difference map and refined using a riding model with C—H = 0.93–0.97 Å, and with *U*_iso_ = 1.2*U*_eq_(C).

### Crystal data

**Table:**

C_21_H_19_N_3_O	*F*(000) = 1392
*M**_r_* = 329.39	*D*_x_ = 1.243 Mg m^−^^3^
Monoclinic, *C*2/*c*	Mo *K*α radiation, λ = 0.71073 Å
Hall symbol: -C 2yc	Cell parameters from 25 reflections
*a* = 12.8122 (10) Å	θ = 10–15°
*b* = 13.9317 (12) Å	µ = 0.08 mm^−^^1^
*c* = 19.7795 (14) Å	*T* = 300 K
β = 94.647 (3)°	Block, yellow
*V* = 3519.0 (5) Å^3^	0.13 × 0.10 × 0.08 mm
*Z* = 8	

### Data collection

**Table:**

Bruker X8 KappaCCD APEXII diffractometer	3220 independent reflections
Radiation source: fine-focus sealed tube	2555 reflections with *I* > 2σ(*I*)
Graphite monochromator	*R*_int_ = 0.032
ω and φ scans	θ_max_ = 25.4°, θ_min_ = 3.6°
Absorption correction: multi-scan (*SADABS*; Sheldrick, 1996)	*h* = −15→15
*T*_min_ = 0.990, *T*_max_ = 0.994	*k* = −16→13
21544 measured reflections	*l* = −23→23

### Refinement

**Table:**

Refinement on *F*^2^	Primary atom site location: structure-invariant direct methods
Least-squares matrix: full	Secondary atom site location: difference Fourier map
*R*[*F*^2^ > 2σ(*F*^2^)] = 0.038	Hydrogen site location: difference Fourier map
*wR*(*F*^2^) = 0.099	H-atom parameters constrained
*S* = 1.02	*w* = 1/[σ^2^(*F*_o_^2^) + (0.0423*P*)^2^ + 1.6343*P*] where *P* = (*F*_o_^2^ + 2*F*_c_^2^)/3
3220 reflections	(Δ/σ)_max_ < 0.001
228 parameters	Δρ_max_ = 0.16 e Å^−^^3^
0 restraints	Δρ_min_ = −0.14 e Å^−^^3^

### Special details

**Table:**

Geometry. All e.s.d.'s (except the e.s.d. in the dihedral angle between two l.s. planes) are estimated using the full covariance matrix. The cell e.s.d.'s are taken into account individually in the estimation of e.s.d.'s in distances, angles and torsion angles; correlations between e.s.d.'s in cell parameters are only used when they are defined by crystal symmetry. An approximate (isotropic) treatment of cell e.s.d.'s is used for estimating e.s.d.'s involving l.s. planes.
Refinement. Refinement of *F*^2^ against ALL reflections. The weighted *R*-factor *wR* and goodness of fit *S* are based on *F*^2^, conventional *R*-factors *R* are based on *F*, with *F* set to zero for negative *F*^2^. The threshold expression of *F*^2^ > σ(*F*^2^) is used only for calculating *R*-factors(gt) *etc*. and is not relevant to the choice of reflections for refinement. *R*-factors based on *F*^2^ are statistically about twice as large as those based on *F*, and *R*- factors based on ALL data will be even larger.

### Fractional atomic coordinates and isotropic or equivalent isotropic displacement parameters (Å^2^)

**Table:**

	*x*	*y*	*z*	*U*_iso_*/*U*_eq_	
C1	0.23744 (12)	−0.00078 (11)	0.47258 (7)	0.0493 (4)	
H1A	0.1759	−0.0412	0.4656	0.059*	
H1B	0.2886	−0.0345	0.5026	0.059*	
C2	0.20897 (11)	0.08866 (12)	0.50454 (8)	0.0500 (4)	
C3	0.18446 (14)	0.15873 (15)	0.53145 (9)	0.0687 (5)	
H3	0.1649	0.2146	0.5529	0.082*	
C4	0.27086 (11)	−0.06084 (10)	0.36193 (7)	0.0418 (3)	
C5	0.30050 (10)	−0.04291 (10)	0.29321 (7)	0.0387 (3)	
C6	0.27571 (10)	0.05269 (10)	0.26392 (7)	0.0396 (3)	
C7	0.19842 (11)	0.05802 (11)	0.20333 (7)	0.0449 (4)	
C8	0.20304 (13)	0.13347 (11)	0.15770 (8)	0.0545 (4)	
H8	0.2563	0.1788	0.1638	0.065*	
C9	0.12883 (16)	0.14127 (14)	0.10342 (9)	0.0710 (5)	
H9	0.1331	0.1913	0.0726	0.085*	
C10	0.04882 (16)	0.07593 (18)	0.09453 (10)	0.0811 (6)	
H10	−0.0017	0.0823	0.0583	0.097*	
C11	0.04357 (15)	0.00075 (17)	0.13950 (11)	0.0806 (6)	
H11	−0.0107	−0.0436	0.1336	0.097*	
C12	0.11855 (13)	−0.00900 (13)	0.19330 (9)	0.0603 (4)	
H12	0.1155	−0.0606	0.2229	0.072*	
C13	0.37939 (11)	0.13592 (10)	0.34662 (7)	0.0418 (3)	
C14	0.46264 (12)	0.20107 (11)	0.34809 (9)	0.0555 (4)	
H14	0.4713	0.2385	0.3100	0.067*	
C15	0.53236 (12)	0.21109 (13)	0.40479 (10)	0.0630 (5)	
H15	0.5886	0.2532	0.4043	0.076*	
C16	0.51758 (12)	0.15823 (13)	0.46185 (9)	0.0598 (4)	
H16	0.5634	0.1653	0.5005	0.072*	
C17	0.43540 (11)	0.09509 (11)	0.46209 (8)	0.0497 (4)	
H17	0.4257	0.0605	0.5013	0.060*	
C18	0.36619 (10)	0.08181 (10)	0.40482 (7)	0.0396 (3)	
C19	0.33035 (10)	−0.12223 (10)	0.25861 (7)	0.0418 (3)	
H19	0.3208	−0.1804	0.2803	0.050*	
C20	0.40194 (12)	−0.04771 (12)	0.16008 (8)	0.0537 (4)	
H20A	0.4630	−0.0639	0.1374	0.064*	
H20B	0.4175	0.0056	0.1900	0.064*	
H20C	0.3460	−0.0306	0.1271	0.064*	
C21	0.39525 (14)	−0.22348 (12)	0.17186 (9)	0.0653 (5)	
H21A	0.4697	−0.2296	0.1706	0.078*	
H21B	0.3620	−0.2300	0.1268	0.078*	
H21C	0.3701	−0.2727	0.2004	0.078*	
N1	0.28111 (9)	0.01576 (8)	0.40710 (6)	0.0415 (3)	
N2	0.30977 (9)	0.13373 (8)	0.28823 (6)	0.0446 (3)	
N3	0.37076 (9)	−0.12945 (9)	0.19895 (6)	0.0462 (3)	
O1	0.23521 (10)	−0.13715 (8)	0.38040 (6)	0.0660 (3)	

### Atomic displacement parameters (Å^2^)

**Table:**

	*U*^11^	*U*^22^	*U*^33^	*U*^12^	*U*^13^	*U*^23^
C1	0.0566 (9)	0.0513 (9)	0.0418 (8)	−0.0016 (7)	0.0155 (7)	0.0015 (7)
C2	0.0463 (8)	0.0615 (10)	0.0434 (8)	0.0012 (7)	0.0107 (6)	−0.0030 (8)
C3	0.0633 (10)	0.0749 (13)	0.0700 (12)	0.0078 (9)	0.0182 (9)	−0.0209 (10)
C4	0.0450 (7)	0.0363 (8)	0.0452 (8)	−0.0025 (6)	0.0109 (6)	0.0006 (6)
C5	0.0420 (7)	0.0362 (7)	0.0384 (7)	−0.0030 (6)	0.0065 (6)	−0.0013 (6)
C6	0.0440 (7)	0.0383 (8)	0.0377 (7)	−0.0002 (6)	0.0114 (6)	−0.0005 (6)
C7	0.0484 (8)	0.0449 (8)	0.0421 (8)	0.0077 (7)	0.0078 (6)	−0.0031 (6)
C8	0.0659 (10)	0.0487 (9)	0.0493 (9)	0.0135 (8)	0.0064 (7)	0.0002 (7)
C9	0.0899 (14)	0.0705 (12)	0.0518 (10)	0.0326 (11)	0.0009 (9)	0.0031 (9)
C10	0.0723 (13)	0.1024 (17)	0.0650 (12)	0.0292 (12)	−0.0170 (10)	−0.0110 (12)
C11	0.0597 (11)	0.0990 (17)	0.0804 (14)	−0.0018 (11)	−0.0105 (10)	−0.0117 (13)
C12	0.0547 (9)	0.0672 (11)	0.0585 (10)	−0.0035 (8)	0.0013 (8)	−0.0025 (8)
C13	0.0461 (8)	0.0360 (8)	0.0448 (8)	−0.0011 (6)	0.0122 (6)	−0.0075 (6)
C14	0.0625 (9)	0.0452 (9)	0.0621 (10)	−0.0125 (7)	0.0257 (8)	−0.0110 (8)
C15	0.0442 (8)	0.0583 (11)	0.0880 (13)	−0.0119 (8)	0.0155 (8)	−0.0270 (10)
C16	0.0444 (8)	0.0629 (11)	0.0711 (11)	0.0035 (8)	−0.0023 (8)	−0.0188 (9)
C17	0.0486 (8)	0.0495 (9)	0.0508 (9)	0.0080 (7)	0.0017 (7)	−0.0060 (7)
C18	0.0390 (7)	0.0369 (7)	0.0440 (8)	0.0019 (6)	0.0098 (6)	−0.0055 (6)
C19	0.0438 (7)	0.0373 (8)	0.0449 (8)	−0.0043 (6)	0.0078 (6)	−0.0009 (6)
C20	0.0531 (9)	0.0621 (10)	0.0480 (9)	−0.0015 (7)	0.0166 (7)	0.0018 (8)
C21	0.0720 (11)	0.0573 (11)	0.0690 (11)	−0.0003 (9)	0.0213 (9)	−0.0224 (9)
N1	0.0481 (6)	0.0402 (7)	0.0375 (6)	−0.0046 (5)	0.0121 (5)	−0.0020 (5)
N2	0.0569 (7)	0.0367 (7)	0.0415 (7)	−0.0019 (5)	0.0111 (5)	0.0003 (5)
N3	0.0501 (7)	0.0442 (7)	0.0458 (7)	−0.0024 (5)	0.0138 (6)	−0.0082 (6)
O1	0.0993 (9)	0.0428 (6)	0.0605 (7)	−0.0180 (6)	0.0342 (6)	−0.0033 (5)

### Geometric parameters (Å, º)

**Table:**

C1—C2	1.457 (2)	C12—H12	0.9300
C1—N1	1.4700 (18)	C13—C18	1.398 (2)
C1—H1A	0.9700	C13—C14	1.399 (2)
C1—H1B	0.9700	C13—N2	1.4012 (18)
C2—C3	1.167 (2)	C14—C15	1.383 (2)
C3—H3	0.9300	C14—H14	0.9300
C4—O1	1.2244 (16)	C15—C16	1.373 (3)
C4—N1	1.3911 (18)	C15—H15	0.9300
C4—C5	1.4622 (19)	C16—C17	1.372 (2)
C5—C19	1.3705 (19)	C16—H16	0.9300
C5—C6	1.4767 (19)	C17—C18	1.393 (2)
C6—N2	1.2893 (17)	C17—H17	0.9300
C6—C7	1.493 (2)	C18—N1	1.4301 (17)
C7—C12	1.388 (2)	C19—N3	1.3303 (18)
C7—C8	1.390 (2)	C19—H19	0.9300
C8—C9	1.380 (2)	C20—N3	1.4482 (19)
C8—H8	0.9300	C20—H20A	0.9600
C9—C10	1.372 (3)	C20—H20B	0.9600
C9—H9	0.9300	C20—H20C	0.9600
C10—C11	1.379 (3)	C21—N3	1.4590 (19)
C10—H10	0.9300	C21—H21A	0.9600
C11—C12	1.381 (2)	C21—H21B	0.9600
C11—H11	0.9300	C21—H21C	0.9600
			
C2—C1—N1	112.02 (12)	C15—C14—C13	121.58 (16)
C2—C1—H1A	109.2	C15—C14—H14	119.2
N1—C1—H1A	109.2	C13—C14—H14	119.2
C2—C1—H1B	109.2	C16—C15—C14	119.29 (15)
N1—C1—H1B	109.2	C16—C15—H15	120.4
H1A—C1—H1B	107.9	C14—C15—H15	120.4
C3—C2—C1	177.97 (17)	C17—C16—C15	120.23 (15)
C2—C3—H3	180.0	C17—C16—H16	119.9
O1—C4—N1	119.51 (13)	C15—C16—H16	119.9
O1—C4—C5	123.84 (13)	C16—C17—C18	121.35 (15)
N1—C4—C5	116.61 (12)	C16—C17—H17	119.3
C19—C5—C4	115.50 (12)	C18—C17—H17	119.3
C19—C5—C6	126.24 (12)	C17—C18—C13	119.11 (13)
C4—C5—C6	117.07 (11)	C17—C18—N1	119.76 (13)
N2—C6—C5	126.02 (12)	C13—C18—N1	121.10 (12)
N2—C6—C7	115.98 (12)	N3—C19—C5	130.42 (13)
C5—C6—C7	117.85 (12)	N3—C19—H19	114.8
C12—C7—C8	119.06 (14)	C5—C19—H19	114.8
C12—C7—C6	120.98 (13)	N3—C20—H20A	109.5
C8—C7—C6	119.89 (14)	N3—C20—H20B	109.5
C9—C8—C7	120.13 (17)	H20A—C20—H20B	109.5
C9—C8—H8	119.9	N3—C20—H20C	109.5
C7—C8—H8	119.9	H20A—C20—H20C	109.5
C10—C9—C8	120.55 (18)	H20B—C20—H20C	109.5
C10—C9—H9	119.7	N3—C21—H21A	109.5
C8—C9—H9	119.7	N3—C21—H21B	109.5
C9—C10—C11	119.76 (18)	H21A—C21—H21B	109.5
C9—C10—H10	120.1	N3—C21—H21C	109.5
C11—C10—H10	120.1	H21A—C21—H21C	109.5
C10—C11—C12	120.28 (19)	H21B—C21—H21C	109.5
C10—C11—H11	119.9	C4—N1—C18	120.39 (11)
C12—C11—H11	119.9	C4—N1—C1	115.01 (11)
C11—C12—C7	120.19 (18)	C18—N1—C1	118.30 (11)
C11—C12—H12	119.9	C6—N2—C13	120.00 (12)
C7—C12—H12	119.9	C19—N3—C20	123.73 (12)
C18—C13—C14	118.40 (14)	C19—N3—C21	120.29 (13)
C18—C13—N2	123.75 (12)	C20—N3—C21	115.74 (12)
C14—C13—N2	117.69 (13)		
			
O1—C4—C5—C19	−27.5 (2)	C16—C17—C18—C13	−1.7 (2)
N1—C4—C5—C19	154.90 (12)	C16—C17—C18—N1	−179.66 (13)
O1—C4—C5—C6	140.80 (15)	C14—C13—C18—C17	0.53 (19)
N1—C4—C5—C6	−36.78 (17)	N2—C13—C18—C17	−174.84 (12)
C19—C5—C6—N2	−133.01 (15)	C14—C13—C18—N1	178.43 (12)
C4—C5—C6—N2	60.09 (18)	N2—C13—C18—N1	3.1 (2)
C19—C5—C6—C7	51.59 (19)	C4—C5—C19—N3	−172.74 (14)
C4—C5—C6—C7	−115.32 (14)	C6—C5—C19—N3	20.2 (2)
N2—C6—C7—C12	−146.20 (14)	O1—C4—N1—C18	146.04 (14)
C5—C6—C7—C12	29.67 (19)	C5—C4—N1—C18	−36.26 (18)
N2—C6—C7—C8	30.81 (19)	O1—C4—N1—C1	−5.6 (2)
C5—C6—C7—C8	−153.32 (13)	C5—C4—N1—C1	172.08 (12)
C12—C7—C8—C9	0.2 (2)	C17—C18—N1—C4	−121.72 (14)
C6—C7—C8—C9	−176.90 (14)	C13—C18—N1—C4	60.39 (18)
C7—C8—C9—C10	1.2 (3)	C17—C18—N1—C1	29.02 (18)
C8—C9—C10—C11	−1.2 (3)	C13—C18—N1—C1	−148.87 (13)
C9—C10—C11—C12	−0.1 (3)	C2—C1—N1—C4	−155.14 (13)
C10—C11—C12—C7	1.4 (3)	C2—C1—N1—C18	52.58 (17)
C8—C7—C12—C11	−1.5 (2)	C5—C6—N2—C13	2.0 (2)
C6—C7—C12—C11	175.58 (15)	C7—C6—N2—C13	177.51 (11)
C18—C13—C14—C15	1.4 (2)	C18—C13—N2—C6	−45.70 (19)
N2—C13—C14—C15	177.09 (14)	C14—C13—N2—C6	138.91 (14)
C13—C14—C15—C16	−2.2 (2)	C5—C19—N3—C20	6.6 (2)
C14—C15—C16—C17	1.0 (2)	C5—C19—N3—C21	−179.16 (15)
C15—C16—C17—C18	1.0 (2)		

### Hydrogen-bond geometry (Å, º)

**Table:**

*D*—H···*A*	*D*—H	H···*A*	*D*···*A*	*D*—H···*A*
C15—H15···O1^i^	0.93	2.50	3.415 (2)	170

Symmetry code: (i) *x*+1/2, *y*+1/2, *z*.
